# Factors Predicting Tongue Pressure Decline among Community-Dwelling Older Adults: The Takashimadaira Study

**DOI:** 10.3390/ijerph19137850

**Published:** 2022-06-26

**Authors:** Chika Takahashi, Masanori Iwasaki, Keiko Motokawa, Yutaka Watanabe, Misato Hayakawa, Yurie Mikami, Maki Shirobe, Hiroki Inagaki, Ayako Edahiro, Yuki Ohara, Hirohiko Hirano, Shoji Shinkai, Shuichi Awata

**Affiliations:** 1 Department of Oral Health Sciences for Community Welfare, Graduate School of Medical and Dental Sciences, Tokyo Medical and Dental University, Tokyo 113-8549, Japan; kurumikohe@gmail.com; 2 Tokyo Metropolitan Institute of Gerontology, Tokyo 173-0015, Japan; kikiki_1004@yahoo.co.jp (K.M.); ywata@den.hokudai.ac.jp (Y.W.); erurinrun0424@gmail.com (M.H.); ega0dm@gmail.com (Y.M.); mashirobe@gmail.com (M.S.); inagaki@tmig.or.jp (H.I.); aedahiro514@gmail.com (A.E.); yohara@tmig.or.jp (Y.O.); h-hiro@gd5.so-net.ne.jp (H.H.); sshinkai@tmig.or.jp (S.S.); awata@tmig.or.jp (S.A.); 3 Gerodontology, Department of Oral Health Science, Faculty of Dental Medicine, Hokkaido University, Sapporo 060-8586, Japan; 4 Faculty of Nutrition, Kagawa Nutrition University, Saitama 350-0288, Japan

**Keywords:** epidemiology, geriatrics, longitudinal study, oral health

## Abstract

A limited number of longitudinal studies have explored factors contributing to decreases in tongue pressure (TP). This longitudinal study aimed to clarify the factors affecting TP decline among community-dwelling older adults. We followed the Takashimadaira Study participants with a baseline TP ≥ 30 kPa for 2 years. A TP of <30 kPa at follow-up was defined as TP decline. We used Poisson regression with robust standard errors to explore the factors related to TP decline. The studied baseline variables were dental status, sociodemographic characteristics, health behaviors, appetite, medical conditions, physical function, cognitive status, and anthropometric and body composition characteristics. Inverse probability weighting (IPW) was used to adjust for selection bias. Overall, 357 individuals (159 men and 198 women) with a mean (standard deviation) age of 75.9 (4.1) years were included in the analyses. Of these, 59 study participants (16.5%) exhibited TP decline. After adjusting for baseline TP and applying IPW, poor appetite (incident rate ratio [95% confidence interval] = 1.58 [1.01–2.48]), low skeletal muscle mass index (1.66 [1.02–2.70]), and cognitive impairment (1.93 [1.12–3.33]) were associated with TP decline. In conclusion, we demonstrated that baseline appetite, body composition, and cognitive status could predict future TP decline among community-dwelling older adults.

## 1. Introduction

Tongue pressure (TP) measurement is a quantitative evaluation of tongue function [1]. Low TP in old age leads to swallowing disorders [2], which can cause undernutrition [3]. Furthermore, a previous study demonstrated that TP was associated with increased risks of adverse health outcomes, including sarcopenia and frailty, among community-dwelling older adults [4]. These findings highlight the importance of maintaining adequate TP for longevity. Clarifying the factors related to TP decline in older adults will lead to early detection of those at risk for adverse health events. To date, several health characteristics, including the number of teeth, grip strength, back muscle strength, and sarcopenia, are associated with TP [5,6,7,8,9]. Another study demonstrated that social environment was associated with TP [6]. Although these are important findings, temporal associations cannot be inferred because of the cross-sectional design of these studies. Longitudinal studies are necessary to explore the factors predicting TP decline. Findings based on longitudinal studies can serve as a basis for proposing new strategies against TP-related adverse health outcomes, such as swallowing disorders and undernutrition, through appropriate intervention.

Here, we followed a cohort of community-dwelling older adults for 2 years. The aim of this longitudinal study was to clarify the factors related to TP decline in this population.

## 2. Materials and Methods

### 2.1. Study Population

The present study used data from the Takashimadaira Study. The details of the sampling methodology and selection of the Takashimadaira Study were published previously [10]. The research protocol of the Takashimadaira Study was approved by the Ethics Committee of the Tokyo Metropolitan Institute of Gerontology (approval numbers: 9 and 31 in 2016). Written informed consent was obtained from all participants.

In 2016, TP measurements; assessments of dental status, physical function, and cognitive status; anthropometric and body composition measurements; a questionnaire survey; and a medical interview were conducted for residents aged 70 years or older in the Takashimadaira area, Tokyo, Japan. In 2018, follow-up investigations to measure TP were conducted.

For the current analyses, the inclusion criteria were individuals who were Takashimadaira Study participants and who participated in the 2-year follow-up TP measurement. The exclusion criteria were individuals who had a TP < 30 kPa at baseline [11], those who were lost for follow-up, and those who had incomplete data.

This study is a secondary analysis of the existing dataset of the Takashimadaira Study, and no prestudy power analysis was performed.

### 2.2. TP Measurement

TP was measured at baseline (2016) and at the 2-year follow-up (2018) by qualified survey staff. The authors (H.H., M.S., and Y.O.) provided the staff with 2 h worth of instructions regarding appropriate TP measuring methods [1,5,12].

**Figure 1:**
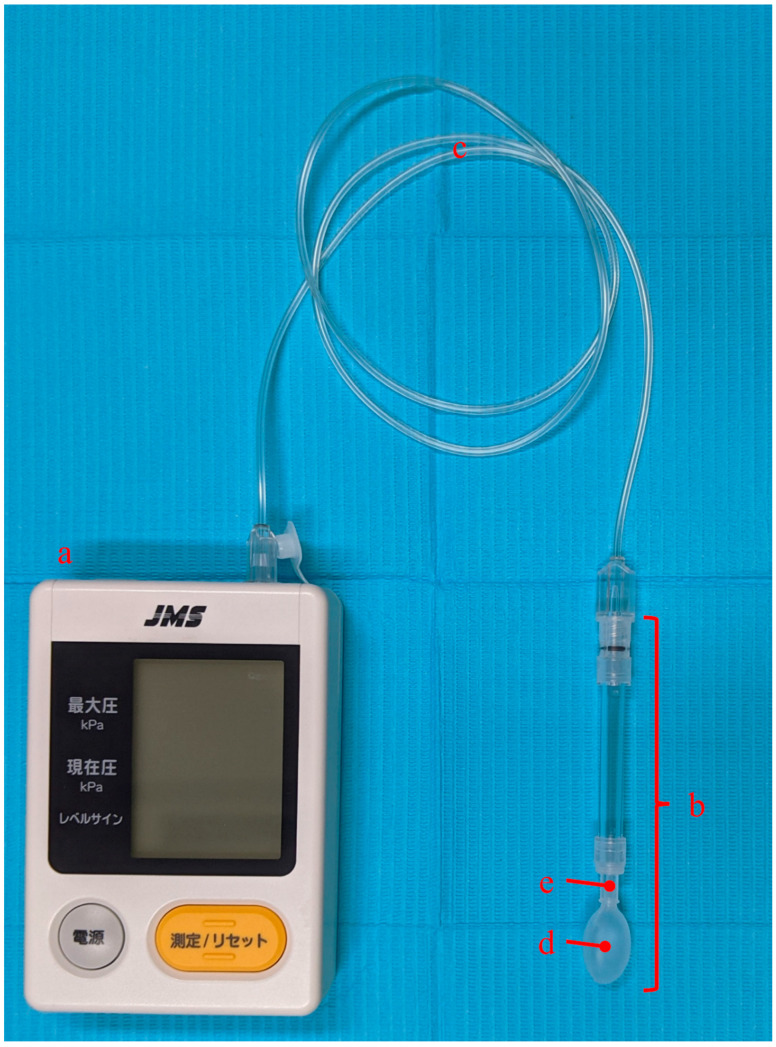
JMS tongue pressure measuring device (TPM-01, JMS Co., Ltd., Hiroshima, Japan). **a**: main body, **b**: probe, **c**: connecting tube, **d**: balloon, **e**: plastic cylinder.

A Tongue Pressure Device (TPM-01, JMS Co., Ltd., Hiroshima, Japan) [1] was used for TP measurement. This device consists of a main body (Figure 1a), probe (Figure 1b), and connecting tube (Figure 1c). A balloon (Figure 1d) is attached to the tip of the probe. The base of the balloon is connected to a plastic cylinder (Figure 1e) that allows the participants to hold the probe in their mouths with their lips closed. The participants sat in a relaxed position and raised their tongue to compress the balloon against the hard palate for 7 s with maximum voluntary effort [1,5]. The device recorded the pressure the balloon received in kPa as the TP. If the participants used dentures, they kept their dentures in their mouth during the measurement [12]. The average TP value, calculated using three measurements with 1 min intervals, was used for the analysis [5].

According to the criteria proposed by the Japanese Society of Gerodontology (JSG) [11], a TP cutoff of 30 kPa was used in the current analysis. We followed the participants whose TP was ≥30 kPa at baseline. A TP of <30 kPa at follow-up was defined as TP decline.

### 2.3. Assessment of Dental Status

The number of teeth and denture use was determined by trained dental professionals. The number of natural teeth was defined as the number of remaining teeth, excluding residual roots. The posterior occlusal support zone (range: 0–4 zones) based on occluding pairs of posterior teeth by using the Eichner classification was counted. The participants were categorized into Eichner group A (having 4 zones), B (having 1–3 zones), or C (having no zone). The number of functional teeth, defined as the number of natural teeth, artificial teeth in dentures, pontics on bridges, and implants [13], was also determined during the assessment.

### 2.4. Anthropometric and Body Composition Measurements

Height and weight were obtained while the participant was wearing light clothing and no shoes. Underweight was defined as a body mass index (BMI) (the weight in kilograms divided by the square of the height in meters) of <18.5. Skeletal muscle mass was measured using the body composition analyzer (InBody S10, Biospace Co., Ltd., Seoul, Korea). The skeletal muscle mass index (SMI) was calculated as the appendicular muscle mass in kilograms divided by the square of the height in meters. An SMI of <7.0 kg/m^2^ in men and <5.7 kg/m^2^ in women was defined as low [14].

### 2.5. Assessments of Physical Function

Handgrip strength was assessed using Smedley-type hand dynamometers (Model YD-100; Yagami Ltd., Tokyo, Japan). The participants performed two trials with the dominant hand, and the better result was used for the analyses. A handgrip strength of <28 kg in men and < 18 kg in women was defined as low [14]. Usual gait speed was measured over 5 m, with acceleration and deceleration phases of 3 m each. This measurement was performed once. A usual gait speed of <1.0 m/s was defined as low [14].

### 2.6. Cognitive Assessment

The Mini-Mental State Examination-Japanese (MMSE-J) [15] was used for cognitive assessment and was performed by trained clinical psychologists and nurses. Cognitive impairment was defined as an MMSE-J score of ≤23.

### 2.7. Questionnaire Survey

Data on participants’ age, sex, educational status (i.e., years of schooling), income, alcohol consumption, smoking status, physical activity level, appetite, social relationships, living situation, and depressive symptoms were obtained using a self-administered questionnaire. Subsequently, these variables were categorized as follows: income was dichotomized as an annual income of <3 million Japanese yen or otherwise (27,312 USD at the 2016 yearly average exchange rate); alcohol consumption was defined by a participant being a daily drinker or otherwise; smoking status was defined by a participant being a current smoker or otherwise. A low physical activity level was defined if participants answered “no” to the following two questions: ‘Do you engage in moderate levels of physical exercise or sports aimed at health?’ and ‘Do you engage in low levels of physical exercise aimed at health?’ [16]. The Council on Nutritional Appetite Questionnaire (CNAQ) was used to assess appetite. Study participants with a CNAQ score ≤ 28 were defined as having poor appetite [17]. Social isolation was identified if participants answered “no” to the following question: ’Do you have contact at least once a week with anyone, including relatives living apart, friends, and neighbors?’ [18]. The Japanese version of the 15-item Geriatric Depression Scale (GDS-15) was used to assess depressive symptoms. Study participants with a GDS-15 score ≥ 6 were defined as having depressive symptoms [19].

### 2.8. Medical Interviews

The data on the comorbidity status (hypertension, heart disease, stroke, and diabetes mellitus) and the use of medication were obtained through a medical interview conducted by well-trained study staff. Study participants with the concurrent use of ≥5 medications were defined as a participant with polypharmacy [20].

### 2.9. Statistical Analyses

Analyses were performed with the statistical software package SPSS statistics version 28.0 (IBM Corp., Armonk, NY, USA). The level of significance (two-tailed test) was set to 0.05.

Student’s *t*-test or the Mann–Whitney *U* test for continuous variables and the chi-squared test for categorical variables were used to compare the baseline study characteristics between men and women.

Furthermore, baseline characteristics were compared between individuals who did and did not (lost for follow-up) participate in the follow-up investigations. Inverse probability weighting (IPW) was calculated using baseline characteristics that showed significant differences between the groups. To adjust for selection bias due to loss to follow-up [21], IPW was applied to all models.

Factors related to TP decline were explored using Poisson regression with robust standard errors. According to previous studies [5,22,23,24], the following baseline variables were considered to be potential factors: dental status, age, sex, educational status, income, alcohol consumption, smoking status, social relationships, living situation, appetite, anthropometric and body composition characteristics, physical function, physical activity level, comorbidities, depressive symptoms, cognitive status, and polypharmacy.

First, we estimated incidence rate ratios (IRRs) and their 95% confidence intervals (CIs) for each baseline variable in the model, adjusting only for baseline TP. Then, a multivariable model was constructed with baseline variables that were significantly associated with TP decline in the initial models. Furthermore, we constructed a multivariable model in which age and sex were incorporated into the model. All models were adjusted for baseline TP because we assumed baseline TP was associated with future TP decline. We also constructed a multivariable model that did not include baseline TP.

## 3. Results

**Figure 2:**
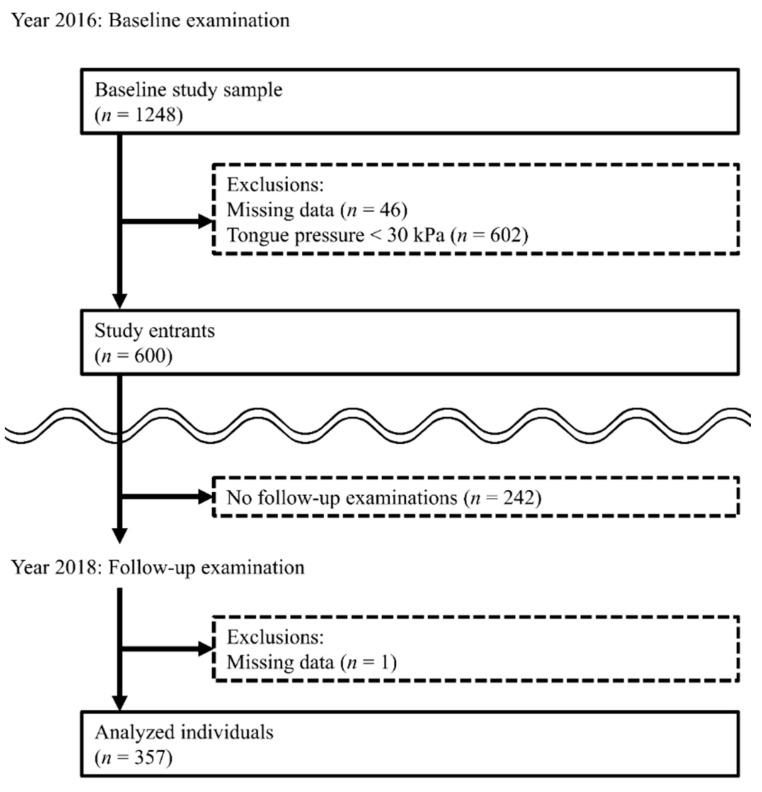
Flow diagram of our study.

The study population consisted of 357 individuals. The selection process results were as follows (Figure 2).

One thousand two hundred and forty-eight individuals participated in the baseline examination in 2016. Of the baseline study sample, 602 individuals who had low TP and 46 individuals who had incomplete data were excluded. Two hundred and forty-two of the 600 study entrants did not participate in the follow-up examination in 2018. Statistically significant differences were observed between the individuals who did (*n* = 358) and those who did not (*n* = 242) participate in the follow-up examinations for the following variables: the number of natural teeth, appetite, grip strength, usual gate speed, and depressive symptoms (Table S1). These variables were used in the IPW calculations. Of the 358 individuals who participated in follow-up examinations, one had missing data and was excluded. Therefore, 357 individuals (159 men and 198 women) with a mean (standard deviation (SD)) age of 75.9 (4.1) years were included in the analyses.

Table 1 shows the baseline characteristics according to sex. Compared to women, men had higher TP values (mean (SD) TP 36.2 (5.1) kPa in women vs. 37.5 (5.9) kPa in men); were more likely to use dentures (42.4% of women vs. 59.1% of men); had a higher educational status (median (interquartile range) years of schooling, 12 (12–14) among women vs. 13 (12–16) among men); were more likely to have diabetes (10.6% of women vs. 18.2% of men); were more likely to be daily drinkers (5.1% of women vs. 27.0% of men), current smokers (2.0% of women vs. 15.1% of men), socially isolated (22.7% of women vs. 56.0% of men), and physically inactive (8.1% of women vs. 15.1% of men); and were less likely to have a low annual income (68.2% of women vs. 46.5% of men) and low SMI (33.3% of women vs. 23.3% of men).

The mean (SD) TP values at the two-year follow-up examination were 35.7 (6.7), 35.6 (6.4), and 35.8 (7.0) in the total population, women, and men, respectively. In total, 59 of the 357 study participants (16.5%) exhibited TP decline. Table 2 shows the results of Poisson regression analyses for TP decline in relation to study participants’ baseline characteristics. After adjusting for baseline TP and applying IPW, poor appetite (IRR = 1.57, 95% CI = 1.001–2.46), low SMI (IRR = 1.67, 95% CI = 1.06–2.63), and cognitive impairment (IRR = 2.22, 95% CI = 1.38–3.57) were significantly associated with TP decline.

Table 3 shows the results from the multivariable models. The first column, model 1, shows the results of the regression model, including the variables that showed a significant association with TP decline. Poor appetite (IRR = 1.58, 95% CI = 1.01–2.45), low SMI (IRR = 1.62, 95% CI = 1.02–2.59), and cognitive impairment (IRR = 1.88, 95% CI = 1.16–3.04) were significantly associated with TP decline. The second column, model 2, shows the model results when adding age and sex as covariates. Poor appetite, low SMI, and cognitive impairment remained statistically significant. The multivariable-adjusted IRRs (95% CI) were 1.58 (1.01–2.48) for poor appetite, 1.66 (1.02–2.70) for low SMI, and 1.93 (1.12–3.33) for cognitive impairment.

The multivariable model not adjusting for baseline TP is presented in Table S2. Poor appetite (IRR = 1.61, 95% CI = 1.01–2.57), low SMI (IRR = 1.89, 95% CI = 1.19–3.01), and cognitive impairment (IRR = 2.98, 95% CI = 1.71–5.18) were consistently and significantly associated with TP decline.

## 4. Discussion

In this 2-year longitudinal study, we explored the baseline factors related to future TP decline among community-dwelling adults aged ≥ 70 years. As a result, poor appetite, low SMI, and cognitive impairment at baseline were found to be associated with TP decline.

The findings of our study were consistent with those of a previous cross-sectional study, in which sarcopenia was associated with decreased TP in older adults [5]. The oral cavity is an organ of one’s body system. Etiological factors for the generalized loss of muscle mass may also affect orofacial muscles, including the tongue muscles, and consequent TP decline. Our hypothesized speculation, as described above, is in agreement with that of earlier studies [25,26,27], where a hypothesized pathway in which a generalized loss of muscle mass and function is associated with a loss of swallowing muscle mass has been proposed. Furthermore, associations of generalized muscle mass with masseter muscle mass, strength, and function have been observed in previous studies [28,29,30,31].

Poor appetite leads to decreased food intake. A previous cross-sectional study demonstrated that older adults with poor appetites are less likely to consume vegetables, fruit, meat, fish, and eggs [32]. Dietary patterns characterized by lower energy, food group (vegetables and fruit), and nutrient (protein and fiber) intakes in community-dwelling older adults with poor appetite have been reported [33]. On the other hand, cognitive impairment can lead to altered appetite, disturbances in meal regularity and a balanced diet, and deterioration of cooking skills and portion control [34,35,36,37], which ultimately leads to unfavorable effects on dietary intake and nutritional status. Previous studies have demonstrated that cognitively impaired older adults were more likely to have poorer nutritional status [38,39]. Poor nutritional status leads to loss of muscle mass among older adults [40,41,42,43,44]. As discussed earlier, orofacial muscles, including the tongue muscles, may be deteriorated by the generalized loss of muscle mass. Overall, we can assume that poor dietary intake and nutritional status caused by poor appetite and/or cognitive impairment leads to TP decline through the generalized loss of muscle mass. Another potential explanation for the fact that poor appetite and cognitive impairment were associated with TP decline is related to dietary status. Decreased food intake caused by poor appetite and/or cognitive impairment can lead to a decrease in the level and duration of perioral muscle activity during the meal, resulting in TP decline. Further studies with information on food intake volume are needed.

As discussed above, it is biologically plausible that poor appetite, low SMI, and cognitive impairment were associated with future TP decline. However, poor appetite, low SMI, and cognitive impairment may affect each other and be related to TP decline in a complex manner. It should be noted that this complex relationship could not be evaluated in the current analysis.

Our study found that dental variables, including numbers of natural and functional teeth, occlusal status, and denture use, were not associated with future TP decline. These findings were consistent with a recent cross-sectional study involving over 5000 community-dwelling older adults [24]. These findings indicate that TP is affected more strongly by muscle status and systemic conditions than morphology in the oral cavity.

The JSG defined oral hypofunction as a seven-component clinical phenotype related to oral functions [11]. TP is one of the evaluation items for oral hypofunction, and a TP value of 30 kPa was set as the cutoff for an oral hypofunction diagnosis. Oral hypofunction has been reported to be associated with undernutrition, sarcopenia, and frailty [45,46,47]. Considering the clinical relevance of oral hypofunction in geriatric health, we decided to follow the JSG criteria and used a TP cutoff of 30 kPa. To the best of our knowledge, there are no commonly used criteria (cutoff values) for TP other than what was proposed by the JSG. The JSG-proposed cutoff value is the most widely used in the field of geriatric dentistry in Japan [11]. Because this cutoff value does not consider potential sex differences in TP [22,23,24], this cutoff value might be subject to further deliberation in the future. However, proposing an alternative cutoff value is beyond the scope of this study.

One distinction of this study with regard to previous studies is that we used a longitudinal design whereby we could demonstrate the temporal association of the baseline factors with future TP decline. Another distinguishing factor is that we calculated IPW based on a rich dataset for oral and systemic health. We found that people who did not participate in the follow-up examinations had poorer health status at baseline compared to those who did participate in the study. This selection bias due to loss to follow-up was adjusted by applying IPW [21].

Although the present study provides a novel finding of a longitudinal association between baseline health characteristics and future TP decline in a community-dwelling older population, several limitations merit consideration. First, data on the development of diseases and related treatment during the study period were not obtained; therefore, we could not consider these effects in the statistical model. Second, the study population consisted of older adults who lived in one specific area of Japan and who voluntarily participated in the survey. It is unclear whether our findings can be applied toother groups. Further studies involving broader populations are necessary to test the generalizability of our results.

## 5. Conclusions

The current study demonstrated that poor appetite, low SMI, and cognitive impairment at baseline were related to TP decline at the 2-year follow-up in community-dwelling older adults. These results suggest that taking into consideration not only individuals’ oral health status but also their nutritional, physical, and cognitive status is necessary in TP management and/or in the prevention of future TP decline. To this end, a comprehensive assessment involving collaborations across multiple professions may be effective.

## Figures and Tables

**Table 1:** Baseline characteristics of the study population according to sex.

	Total	Women	Men	
	N = 357	N = 198	N = 159	*p* Value
Oral health status				
Tongue pressure (kPa) *	36.8 (5.5)	36.2 (5.1)	37.5 (5.9)	0.03
n of natural teeth ^†^	23 (13–27)	24 (16–27)	22 (12–27)	0.09
Posterior occlusal support				0.07
Eichner group C	96 (26.9%)	48 (24.2%)	48 (30.2%)	
Eichner group B	102 (28.6%)	51 (25.8%)	51 (32.1%)	
Eichner group A	159 (44.5%)	99 (50.0%)	60 (37.7%)	
Denture use ^‡^	178 (49.9%)	84 (42.4%)	94 (59.1%)	<0.01
n of functional teeth ^†^	28 (27–28)	28 (27–28)	28 (27–28)	0.51
Other characteristics				
Age *	75.9 (4.1)	75.9 (4.1)	75.9 (4.0)	0.89
Educational status (years of schooling) ^†^	12 (12–16)	12 (12–14)	13 (12–16)	<0.01
Annual income < 3 million JPY ^‡^	209 (58.5%)	135 (68.2%)	74 (46.5%)	<0.01
Daily drinker ^‡^	53 (14.8%)	10 (5.1%)	43 (27.0%)	<0.01
Current smoker ^‡^	28 (7.8%)	4 (2.0%)	24 (15.1%)	<0.01
Social isolation ^‡^	134 (37.5%)	45 (22.7%)	89 (56.0%)	<0.01
Living alone ^‡^	128 (35.9%)	97 (49.0%)	31 (19.5%)	<0.01
Poor appetite ^‡^	106 (29.7%)	58 (29.3%)	48 (30.2%)	0.85
Underweight ^‡^	11 (3.1%)	9 (4.5%)	2 (1.3%)	0.07
Low SMI ^‡^	103 (28.9%)	66 (33.3%)	37 (23.3%)	0.04
Low grip strength ^‡^	16 (4.5%)	5 (2.5%)	11 (6.9%)	0.05
Low usual gait speed ^‡^	23 (6.4%)	9 (4.5%)	14 (8.8%)	0.10
Low physical activity level ^‡^	40 (11.2%)	16 (8.1%)	24 (15.1%)	0.04
Comorbidity status ^‡^				
Hypertension	176 (49.3%)	95 (48.0%)	81 (50.9%)	0.58
Heart disease	67 (18.8%)	37 (18.7%)	30 (18.9%)	0.97
Stroke	24 (6.7%)	11 (5.6%)	13 (8.2%)	0.33
Diabetes	50 (14.0%)	21 (10.6%)	29 (18.2%)	0.04
Depressive symptoms ^‡^	89 (24.9%)	51 (25.8%)	38 (23.9%)	0.69
Cognitive impairment ^‡^	17 (4.8%)	9 (4.5%)	8 (5.0%)	0.83
Polypharmacy ^‡^	109 (30.5%)	55 (27.8%)	54 (34.0%)	0.21

* Presented as the mean (SD); ^†^ Presented as the median (IQR); ^‡^ Presented as n (%); IQR, interquartile range; JPY, Japanese yen; SD, standard deviation; SMI, skeletal muscle mass index.

**Table 2:** Tongue pressure decline in relation to study participants’ baseline characteristics.

Variables *	IRRs ^†,‡^	95% CIs	*p* Value
Oral health status			
n of natural teeth (per one tooth increase)	0.99	(0.96–1.01)	0.36
Posterior occlusal support			
Eichner group C	Ref.		
Eichner group B	0.94	(0.52–1.70)	0.84
Eichner group A	0.81	(0.46–1.43)	0.47
Denture use	1.23	(0.79–1.91)	0.35
n of functional teeth (per one tooth increase)	1.01	(0.92–1.10)	0.86
Other characteristics			
Age (per one year increase)	1.01	(0.96–1.07)	0.67
Men (vs. women)	1.03	(0.65–1.62)	0.91
Educational Status (per one year of schooling increase)	0.95	(0.87–1.04)	0.28
Annual income < 3 million JPY	0.86	(0.55–1.35)	0.51
Daily drinker	0.71	(0.33–1.50)	0.36
Current smoker	0.67	(0.24–1.86)	0.44
Social isolation	0.83	(0.51–1.35)	0.45
Living alone	1.23	(0.78–1.95)	0.37
Poor appetite	1.57	(1.001–2.46)	0.049
Underweight	1.16	(0.33–4.16)	0.82
Low SMI	1.67	(1.06–2.63)	0.03
Low grip strength	1.26	(0.45–3.51)	0.66
Low usual gait speed	1.37	(0.66–2.85)	0.40
Low physical activity level	0.96	(0.48–1.95)	0.92
Comorbidity status			
Hypertension	1.06	(0.68–1.66)	0.80
Heart disease	1.11	(0.63–1.96)	0.71
Stroke	1.06	(0.45–2.46)	0.90
Diabetes	1.16	(0.56–2.41)	0.69
Depressive symptoms	0.95	(0.56–1.62)	0.85
Cognitive impairment	2.22	(1.38–3.57)	<0.01
Polypharmacy	1.26	(0.78–2.05)	0.34

CI, confidence interval; IRR, incidence rate ratio; JPY, Japanese yen; Ref., reference; SMI, skeletal muscle mass index. * Except for numbers of natural teeth and functional teeth, age, sex, and educational status, IRRs and CIs of being positive are presented. ^†^ Applying inverse probability weighting. ^‡^ Adjusting for baseline tongue pressure.

**Table 3:** Multivariable Poisson regression models for the factors related to tongue pressure decline.

	Outcome = Having Tongue Pressure of <30 kPa at 2-Year Follow-Up Assessment					
	Model 1 (Baseline Variables That Yielded *p* Values < 0.05 in the Models Adjusted Only for Baseline Tongue Pressure *)			Model 2 (Model 1 + Age and Sex)		
Variables	IRRs ^†,‡^	95% CIs	*p* Value	IRRs ^†,‡^	95% CIs	*p* Value
Poor appetite	1.58	(1.01–2.45)	0.04	1.58	(1.01–2.48)	0.046
Low SMI	1.62	(1.02–2.59)	0.04	1.66	(1.02–2.70)	0.04
Cognitive impairment	1.88	(1.16–3.04)	0.01	1.93	(1.12–3.33)	0.02
Age (per one-year increase)				0.99	(0.94–1.04)	0.67
Men (vs. women)				1.02	(0.64–1.63)	0.94

CI, confidence interval; IRR, incidence rate ratio; SMI, skeletal muscle mass index. * Candidate baseline variables are presented in Table 2. ^†^ Applying inverse probability weighting. ^‡^ Adjusting for baseline tongue pressure.

## Data Availability

The data presented in this study are available upon request from the corresponding author. The data are not publicly available due to ethical and legal restrictions imposed by the Ethics Committee of the Tokyo Metropolitan Institute of Gerontology.

## Supplementary Materials

The following supporting information can be downloaded at https://www.mdpi.com/article/10.3390/ijerph19137850/s1, Table S1: Baseline characteristics of the study entrants with and without follow-up examinations. Table S2: Multivariable Poisson regression models for the factors related to tongue pressure decline (not considering baseline tongue pressure level).
