# Supplementary material for: Factors Predicting Tongue Pressure Decline among Community-Dwelling Older Adults: The Takashimadaira Study
Source: Int J Environ Res Public Health. 2022 Jun 26;19(13):7850. doi: 10.3390/ijerph19137850 (PMC9265909; doi:10.3390/ijerph19137850)
Supplement: Supplementary file 1 [file ijerph-19-07850-s001.zip › ijerph-1754384-supplementary.pdf]

Table S1. Baseline characteristics of the study entrants with and without follow-up examinations.

	Study Entrants	Follow-Up Examinations		
		Did Not Participate (Dropout)	Participated	
	N = 600	N = 242	N = 358	*p* Value
Oral health status				
Tongue pressure (kPa)*	36.5 (5.2)	36.0 (4.7)	36.8 (5.5)	0.07
n of natural teeth†	22 (12–27)	21 (10–26)	23 (13–27)	0.02
Posterior occlusal support				0.09
Eichner group C	183 (30.5%)	86 (35.5%)	97 (27.1%)	
Eichner group B	162 (27.0%)	60 (24.8%)	102 (28.5%)	
Eichner group A	255 (42.5%)	96 (39.7%)	159 (44.4%)	
Denture use‡	313 (52.2%)	134 (55.4%)	179 (50.0%)	0.20
n of functional teeth†	28 (27–28)	28 (27–28)	28 (27–28)	0.16
Other characteristics				
Age*	76.2 (4.4)	76.6 (4.7)	75.9 (4.1)	0.08
Educational status (years of schooling)†	12 (12–15)	12 (12–14)	12 (12–16)	0.10
Annual income < 3 million JPY‡	365 (60.8%)	156 (64.5%)	209 (58.4%)	0.13
Daily drinker‡	101 (16.8%)	48 (19.8%)	53 (14.8%)	0.11
Current smoker‡	47 (7.8%)	19 (7.9%)	28 (7.8%)	0.99
Social isolation‡	229 (38.2%)	95 (39.3%)	134 (37.4%)	0.65
Living alone‡	234 (39.0%)	105 (43.4%)	129 (36.0%)	0.07
Poor appetite‡	206 (34.3%)	99 (40.9%)	107 (29.9%)	0.01
Low BMI‡	19 (3.2%)	8 (3.3%)	11 (3.1%)	0.87
Low SMI‡	186 (31.0%)	82 (33.9%)	104 (29.1%)	0.21
Low grip strength‡	42 (7.0%)	26 (10.7%)	16 (4.5%)	<0.01
Low usual gait speed‡	56 (9.3%)	32 (13.2%)	24 (6.7%)	0.01
Low physical activity level‡	77 (12.8%)	36 (14.9%)	41 (11.5%)	0.22
Comorbidity status‡				
Hypertension	305 (50.8%)	128 (52.9%)	177 (49.4%)	0.41
Heart disease	107 (17.8%)	40 (16.5%)	67 (18.7%)	0.49
Stroke	44 (7.3%)	20 (8.3%)	24 (6.7%)	0.47
Diabetes	86 (14.3%)	36 (14.9%)	50 (14.0%)	0.76
Depressive symptoms‡	184 (30.7%)	94 (38.8%)	90 (25.1%)	<0.01
Cognitive impairment‡	37 (6.2%)	20 (8.3%)	17 (4.7%)	0.08
Polypharmacy‡	200 (33.3%)	90 (37.2%)	110 (30.7%)	0.10

*Presented as the mean (SD)
†Presented as the median (IQR)
‡Presented as n (%)
BMI, body mass index; IQR, interquartile range; JPY, Japanese yen; SD, standard deviation; SMI, skeletal muscle mass index.

Table S2. Multivariable Poisson regression models for the factors related to tongue pressure decline (not considering baseline tongue pressure level).

	Outcome = having tongue pressure of <30 kPa at 2-year follow-up assessment		
**Variables**	IRRs*	95% CIs	*p* value
Poor appetite	1.61	(1.01–2.57)	0.047
Low SMI	1.89	(1.19–3.01)	0.01
Cognitive impairment	2.98	(1.71–5.18)	<0.01
Age (per one-year increase)	1.00	(0.95–1.06)	0.93
Men (vs. women)	0.89	(0.55–1.44)	0.63

CI, confidence interval; IRR, incidence rate ratio; SMI, skeletal muscle mass index.
*Applying inverse probability weighting.
